# Vitamin D Status in Children with Autism Spectrum Disorders: Determinants and Effects of the Response to Probiotic Supplementation

**DOI:** 10.3390/metabo12070611

**Published:** 2022-07-01

**Authors:** Letizia Guiducci, Cristina Vassalle, Margherita Prosperi, Elisa Santocchi, Maria Aurora Morales, Filippo Muratori, Sara Calderoni

**Affiliations:** 1 Institute of Clinical Physiology, CNR, 56124 Pisa, Italy; letiziag@ifc.cnr.it (L.G.); morales@ifc.cnr.it (M.A.M.); 2 Fondazione CNR-Regione Toscana G. Monasterio, 56124 Pisa, Italy; 3 IRCCS Fondazione Stella Maris, Calambrone, 56128 Pisa, Italy; margherita.prosperi@fsm.unipi.it (M.P.); filippo.muratori@fsm.unipi.it (F.M.); sara.calderoni@fsm.unipi.it (S.C.); 4 UFSMIA Zona Valle del Serchio, Azienda USL Toscana Nord Ovest, 55032 Castelnuovo di Garfagnana, Italy; santocchielisa@gmail.com; 5 Department of Clinical and Experimental Medicine, University of Pisa, 56126 Pisa, Italy

**Keywords:** autism spectrum disorder, 25(OH)D, gastrointestinal symptoms, ADOS, leptin, probiotic

## Abstract

A relationship between the presence of clinical symptoms and gastrointestinal (GI) disturbances associated with nutritional deficiencies, including vitamin D (25(OH)D) deficiency, has been observed in autism spectrum disorder (ASD). The aim was to evaluate 25(OH)D levels according to the annual rhythm cycle, gender, the severity of autism, nutritional or clinical status, inflammatory and metabolic biomarkers, GI symptoms, and the clinical response to probiotic/placebo supplementation in preschooler children with ASD. Eighty-one ASD preschoolers (67 males) were assessed with standardized tools for ASD severity (ADOS score) and GI symptoms (by GI-Index at six-items and at nine-items, the latter defined as the Total GI-Index). The 25(OH)D levels were compared among different ASD subgroups according to metabolic and inflammatory biomarkers (leptin, insulin, resistin, PAI-1, MCP-1, TNF-alfa, and IL-6), gender, and the presence or absence of: (i) GI symptoms, (ii) the response to probiotic supplementation (the improvement of GI symptomatology), (iii) the response to probiotic supplementation (improvement of ASD severity). Only 25% of the ASD children presented an adequate 25(OH)D status (≥30 ng/mL according to the Endocrine Society guidelines). All the 25(OH)D levels falling in the severe deficiency range (<10 ng/mL) were observed in the male subgroup. A significant inverse correlation between 25(OH)D and leptin was observed (R = −0.24, *p* = 0.037). An inverse correlation was found between 25(OH)D levels and the GI Index 6-Items and Total GI-Index (R = −0.25, *p* = 0.026; −0.27, = 0.009) and a direct relationship with the probiotic response (R = 0.4, *p* = 0.05). The monitoring of 25(OH)D levels and the co-administration of 25(OH)D and probiotic supplementation could be considered in ASD from early ages.

## 1. Introduction

Autism spectrum disorders (ASD) are neurodevelopmental disorders characterized by persistent social communication difficulties with concurrent restricted interests, repetitive activities, and sensory abnormalities [1]. The pathogenesis of ASD is complex and not yet fully clarified, but it is widely recognized that genetic liability and environmental factors interact in producing the early alteration of structural and functional brain development, responsible for ASD symptoms [2]. Emerging evidence indicates that gestational or developmental vitamin D (25(OH)D) deficiency may be associated with an increased ASD risk, likely due to its known pleiotropic effects, including those on the central nervous system. The 25(OH)D role in brain development and numerous neuronal functions goes well beyond the traditional and well recognized effects on the skeletal system and mineral metabolism [3,4]. Experimental studies have shown the several neurotrophic and neuroprotective effects of 25(OH)D vitamin, its anti-inflammatory and antioxidant actions (e.g., the modulation of cytokine levels), its role on intracellular calcium regulation and on the dopaminergic system, neurotransmission, and neuroplasticity, as well as the modulation of neurogenesis, apoptosis, and mitosis neuronal differentiation, structure, and metabolism [5]. For these reasons, 25(OH)D has been studied for its relationship to psychiatric diseases such as attention deficit hyperactivity disorder and schizophrenia [6,7]. Similarly, 25(OH)D deficiency could play a role in the etiology of ASD by exerting effects on various pathways involved in brain development and in the balance of neurotransmitters in the brain, by reducing antioxidant and immunological responses [8]. Accordingly, the role of 25(OH)D in ASD pathophysiology was proposed for the first time by Cannel in 2008 [9]; afterwards, other studies have shown how low 25(OH)D status in gestational and early development represents an environmental risk factor for ASD [10,11]. It is worthy of note that low serum vitamin D levels have been demonstrated to be decreased in ASD patients [12,13]. Recently, the role of hypovitaminosis D in the etiology of gastrointestinal symptoms (GI) commonly associated with ASD has been investigated, and a significant correlation between lower 25(OH)D levels and GI symptomatology was reported [14].

The microbiota–gut–brain axis has been recognized as a key regulator of neuropsychiatric health [15], and probiotics may modulate brain activity and function, possibly improving the behavioral profiles of ASD subjects. Recent data have suggested that 25(OH)D may affect the microbiome by changing its composition and by playing a role in the integrity of the gut epithelial barrier [16]. However, no study as of now has investigated the effects of 25(OH)D status on the response to probiotic supplementation in ASD.

Thus, the current study aims to investigate the role of 25(OH)D status in 81 ASD children, and to assess the correlation between 25(OH)D levels and ASD severity, GI symptomatology, and inflammatory, and metabolic markers. In particular, a panel including biomarkers previously associated with the extent and severity of ASD (leptin, insulin, resistin, plasminogen activator inhibitor 1 (PAI-1), monocyte chemoattractant protein-1 (MCP-1), tumor necrosis factor alpha (TNF-alfa), and interleukin 6 (IL-6) [17,18,19] was evaluated for each patient enrolled. The relationship between 25(OH)D blood levels and GI symptomatology as well as ASD severity after 6 months of probiotic treatment was also analyzed.

## 2. Results

### 2.1. Characteristics of the Population

General and clinical characteristics of 81 participants are reported in Table 1. A higher prevalence of males was observed. We did not find statistically significant differences between GI vs. No-GI group or between females and males as far as age, BMI, ADOS CSS, and the other studied blood parameters.

### 2.2. Annual Rhythm Cycle and Anthropometric Characteristics

**Figure 1:**
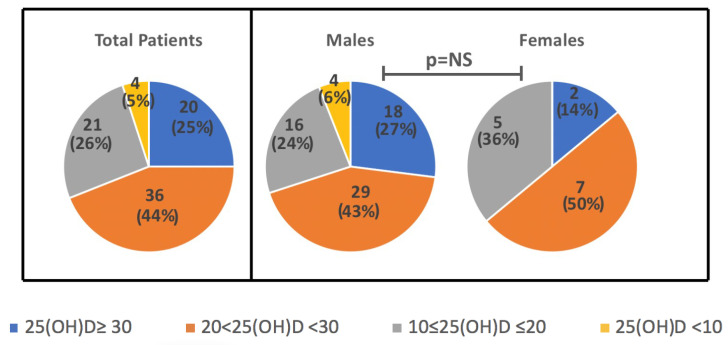
Distribution of 25(OH)D ranges in total sample and in the subgroups of M and F children (ranges: adequate levels ≥30, insufficient 21–29, deficient <20 ng/mL with severe deficiency for values <10 ng/mL). Data are N (%).

Levels of 25(OH)D did not differ when considered according to day saving time-DST (26.6 ± 8.9 vs 22.8 ± 10.7 ng/mL, in DST vs no-DST, *p* = NS). However, when seasonality was considered, 25(OH)D levels were slightly higher in summer/autumn (28.5 ± 8.3 ng/mL), as compared to spring/winter (21.1 ± 7.0 ng/mL), although values did not reach a statistically significant difference. There was no seasonal difference in levels based on the gender (*p* = 0.6), as well as no significant differences in mean 25(OH)D between females and males (23.6 ± 7.2 ng/mL vs 25.1 ± 10.5 ng/mL). In Figure 1. the distribution of 25(OH)D ranges according to the Endocrine Society’s guidelines is reported (adequate levels ≥30, insufficient 21–29, deficient <20 ng/mL with severe deficiency for values <10 ng/mL) [20] in the overall population and in the two sexes. To note, all the 25(OH)D levels falling in the severe deficiency range (<10 ng/mL) were observed in the male children subgroup (one taken in Autumn, one in Spring, two in Winter).

### 2.3. 25(OH)D According to Blood Parameters and BMI

A significant inverse relationship (R = −0.24, *p* = 0.037) was shown between 25(OH)D and leptin. We also found a linear regression between leptin levels and BMI (R = 0.34, *p* = 0.002), but not between leptin and the 6GI-Index or Total GI-Index, neither between 25(OH)D and BMI.

### 2.4. 25(OH)D According to GI and ADOS

**Figure 2:**
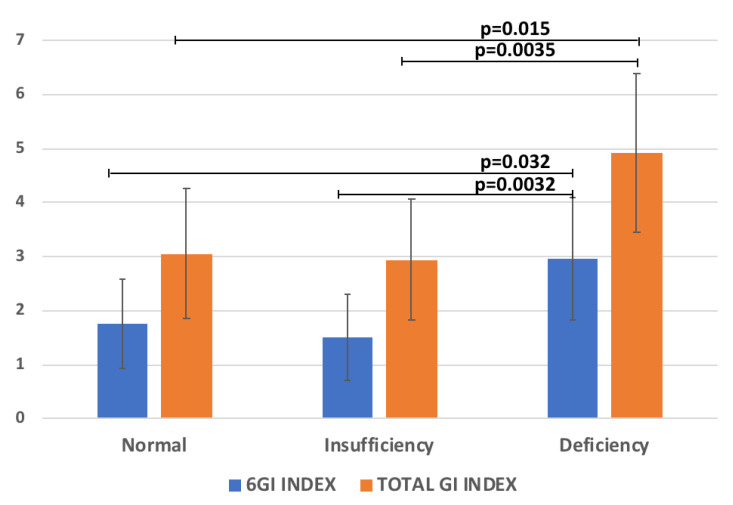
The scores of 6GI-Index and Total GI-Index vs 25(OH)D categories (normal levels ≥30, insufficient 21–29, deficient <20 ng/mL [20].

The regression analysis between 25(OH)D with all the variables reported in Table 1 was performed. Significant relationships between 25(OH)D and the GI Index 6-Items (R = −0.25, *p* = 0.026), and the Total GI-Index severity score (R = −0.27, *p* = 0.009) were found. Accordingly, the levels of the 6GI-Index and Total GI-Index severity score significantly increased according to 25(OH)D reduction (Figure 2). Conversely, no significant correlation with ADOS parameters was found.

### 2.5. Multivariate Regression Analysis for 25(OH)D

A multiple regression analysis was also applied to verify the effect of significant variables (leptin and Total GI severity score,) in determining 25(OH)D concentration. A multiple regression analysis showed that leptin (T-value −2.1, *p* = 0.048), and the Total GI severity score (−2.7, = 0.007) remained as independent determinants affecting 25(OH)D levels in our population.

### 2.6. 25(OH)D According to ADOS Total Score Improvement Due to Probiotics

In the placebo group, there were 9 children in the group with “ADOS Total Score Improved”, 12 in the “ADOS Total Score Unchanged”, and 11 in the “ADOS Total Score Worsened”. These groups did not show any difference in 25(OH)D mean baseline levels (24.2 ± 12.6, 28.1 ± 12.2, and 23.7 ± 7.7 ng/mL in “Improved”, “Unchanged”, and “Worsened” ADOS Total Score, respectively, *p* = ns).

**Figure 3:**
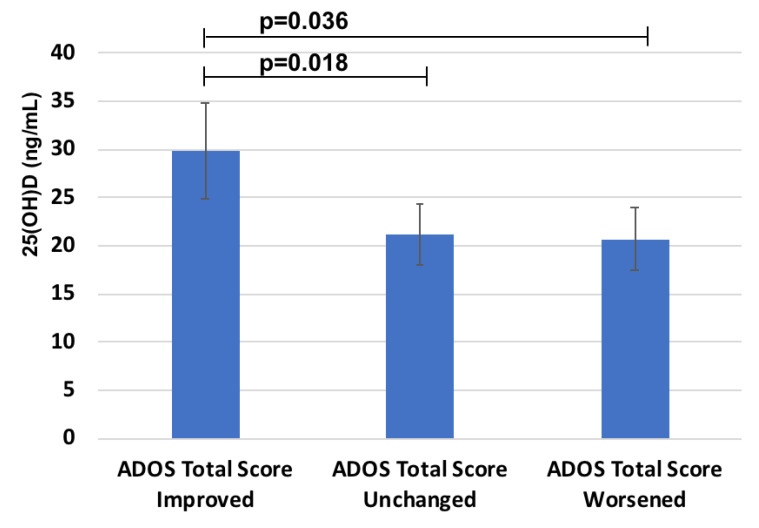
25(OH)D levels in the groups with different response to probiotics treatment on ADOS Total score.

Instead, a significant relationship was found between 25(OH)D and the response to probiotics treatment measured by the decreased ADOS Total score in the probiotic group (n = 31) (R = 0.4, *p* = 0.05). Moreover, when the group treated with probiotic was stratified depending on the different responses measured as delta ADOS Total Score, children in the “ADOS Total Score Improved” group (n = 14) showed the highest 25(OH)D status (Figure 3) (29.9 ± 9.9 versus 21.2 ± 6.3 ng/mL in 11 children of the “Unchanged” group and 20.7 ± 8.8 ng/mL in 6 children belonging to the “Worsened” group, respectively).

Notably, all children with markedly reduced 25(OH)D (<10 ng/mL) were in the group of worsened ADOS Total Score (negative predictive power of 100%).

None of the anthropometric and biochemical variables influenced ADOS Total Score improvement at the univariate analysis except for 25(OH)D. Having 25(OH)D below 30 ng/mL carries a 5.6 higher risk of a lack of improvement in ADOS after 6 months of probiotic supplementation (intervals of confidence 1–35, *p* ≤ 0.05).

### 2.7. 25(OH)D According to GI Improvement Due to Probiotics

When the children, stratified by treatment response, were evaluated for GI symptoms, no significant differences in 25(OH)D values were observed among the three groups treated with probiotic (GI-Index worsened, GI-Index unchanged, and GI-Index improved).

## 3. Discussion

### 3.1. Population Characteristics

Our study population consisted mainly of males, with a ratio between ASD males and ASD females similar to that reported in the literature [21]. The children were all in preschool age as the intent of the original study was to verify the effect of the probiotic on autistic symptoms, hypothesizing that children in their first years of age retain greater neuronal plasticity with neurodevelopmental processes still in progress, and as such they could benefit more from this supplementation [22].

No significant abnormalities in the inflammatory and biomarkers analyzed were observed in the studied population. We previously reported a lack of reference values for non-routine biomarkers such as cytokines, especially in the pediatric range [17]. The results of these biomarkers may vary according to the tests and instrumentation used as well as specimen sampling and storing. Nonetheless, our values are comparable to those reported in the literature for children of comparable age [23,24].

### 3.2. 25(OH)D, Anthropometric Data, and Annual Rhythm Cycle

In recent years, many studies have examined the link between 25(OH)D and ASD by comparing 25(OH) levels of ASD children with controls. The review by Alzghoul reported lower levels of 25(OH)D in ASD as compared to the control samples, with a significant percentage of ASD patients with insufficiency/deficiency [8]. In particular, the percentages of ASD patients with deficient or insufficient vitamin D levels were 86% [25], 87% [26], and 100% [27] compared to typically developing children. In a similar vein, 75% of subjects we examined showed 25(OH)D levels deficiency (under 20 ng/mL) or insufficient (between 21 and 29 ng/mL). Our report did not detect a significant difference in 25(OH)D levels between female and male children as reported also in a previous study [28]. Nonetheless, the fact that in our sample all subjects with 25(OH)D severe deficiency were males, merits further investigation. In fact, the low 25(OH)D levels in pediatric age may further worsen during adolescence, a critical period when the restructuring process of bone development occurs, thus potentially interfering with a proper growth in this age stage. In addition, beyond gender-related differences in 25(OH)D levels, the capacity to utilize 25(OH)D may differ between sexes. Accordingly, Cannel [29] argues that the higher prevalence of ASD in males could be partly related to the fact that 25(OH)D metabolism may markedly differ under the effects of the sex hormones, in particular estrogen, which can enhance the beneficial effects of 25(OH)D on brain development. This consideration is supported by studies showing that the developing brain of a female fetus could more efficiently use available 25(OH)D due to its higher estrogen levels as opposed to the brain of a male fetus, with its higher testosterone levels [30]. In a situation where the levels of 25(OH)D are more than sufficient, the differences due to the distinct actions of the sex hormones could be overcome [29]. On the other hand, a condition of 25(OH)D deficiency, both maternal and in early childhood, could contribute to abnormal brain development favoring ASD onset with a higher incidence in males [11,31]. Accordingly, recent experimental data confirmed that 25(OH)D deficiency increases testosterone levels in maternal blood and male embryonic rat brains [32]. Therefore, a 25(OH)D deficient status could represent a predisposing factor for ASD onset, increasing foetal exposure to testosterone.

The active steroid 25(OH)D is obtained by dietary uptake or mainly synthesized in human skin after exposure to sunlight, and it is known to vary according to seasonality. In our population, no significant differences were found as far as a 25(OH)D annual rhythm cycle, although levels in summer/autumn were higher compared to those taken during spring/winter, suggesting the important contribution of sun exposure to achieve higher 25(OH)D levels, and the importance of outdoor activities in these children. To note, all over the year, the average values remained suboptimal as compared to the recommended level (according to the Endocrine Society’s guidelines) probably due to poor sun exposure. In fact, although ASD children should benefit from sunlight, either the ASD children tend to refuse communal play outdoors or their parents are likely to keep them indoors since they cannot be left alone to play outdoors like typical developing children [28]. In support of the critical role of exposure to sunlight, a significant positive association between latitude and the prevalence of autism has been reported [13] and Grant and coll. [33] found that children who live in low UVB light have almost three times the prevalence of ASD compared to children who live in sunny areas. Moreover, ASD children may show particular dietary habits, often having food selectivity and restricted diets, which expose them to an increased risk for micronutrient deficiencies [34]; thus, 25(OH)D synthesis or intake may be reduced in these children.

Based on these assumptions, the monitoring of vitamin D levels could be considered in autistic children, especially in males, to take protective measures and treat this condition as early as possible.

### 3.3. 25(OH)D, Blood Biomarkers, and BMI

Some studies described that leptin in ASD subjects is higher than in typically developing controls [19,35]. This hormone has an important role in the regulation of food intake and body weight [36], and its expression by adipose tissue is also influenced by feeding behavior [37]. In Castro’s study [38] ASD participants showed higher levels of leptin in comparison with typically developing children, and a positive correlation between leptin and fat mass was demonstrated, bringing out the role of leptin as a marker of adiposity in ASD children. Initially, the adipokines, hormones synthesized mainly by the adipocytes, were associated with eating disorders and obesity but later studies showed their important role in the regulation of immune responses and inflammation; for this reason, their involvement in the pathophysiology of autism was hypothesized [39]. Beyond ASD, an inverse association between leptin levels and 25(OH)D concentration was found in observational studies [40]. A recent review indicates that leptin plays roles in immunity, the regulation of insulin secretion, sex hormone release, performs lipolysis in adipocytes, and modulates plasticity in learning and memory-based behavioral tasks [41]. The presence of leptin receptors in specific regions of the brain implies the potential effect of this hormone in multiple mechanisms related to the function and structure of the brain [42]. In fact, leptin shares structural and functional similarities with several cytokines, many of which are involved in neurodevelopment, including IL-6 and IL-12 [43]. The inverse relationship between leptin and 25(OH)D levels found in our study could be related to the fact that the leptin levels are regulated by 25(OH)D. In particular, 25(OH)D may directly affect the expression of leptin, reducing its release from adipose tissue and consequently decreasing tissue inflammation through the inhibition of NF-kB signaling [38]. It has also recently been demonstrated that 25(OH)D affects brain serotonin concentrations, and may control leptin levels [44]. These interactions could be relevant to neuropsychiatric disorders, such as autism, with a possible impact also on the eating behavior [44].

### 3.4. 25(OH)D, ADOS, and GI

In our sample, no baseline correlation between 25(OH)D and ADOS was observed. In the literature it is widely debated whether 25(OH)D levels correlate with the severity of ASD, with some evidence reporting an inverse relationship between the averaged serum 25(OH)D level and the severity of ASD (*p* > 0.001) [26], and others reporting a lack of correlation [27]. Interestingly, in a recent study [8] no significant correlation was found between vitamin D levels and calcium levels or EEG abnormalities in children with ASD. Therefore, the link between 25(OH)D values and ASD severity remains a topic to be further investigated.

The deficiency of 25(OH)D, which affects approximately 80% of the general population, has been linked with gut dysbiosis and inflammation [45]. In our study the regression analysis between 25(OH)D with GI Index 6-Items and the Total GI severity score showed a significantly negative relationship. In fact, the levels of the 6GI-Index and the Total GI- Index severity score significantly increased according to 25(OH)D reduction (Figure 3). This result is in agreement with a previous study detecting that children with ASD and 25(OH)D deficiency experienced a significantly higher number of GI complaints compared to 25(OH)D-non-deficient children with ASD [14]. Indeed, the authors found an association between low 25(OH)D levels (≤30 ng/mL) and various GI problems, including diarrhea, constipation, pain, and bloating. Interestingly, to corroborate this result, 25(OH)D supplementation was demonstrated to improve the symptoms of GI problems in ASD patients [46].

### 3.5. 25(OH)D and the Effects of Probiotic Supplementation

One significant result that emerged from this study is that ASD children who showed significant improvements in ADOS scores after probiotic supplementation [47], had higher 25(OH)D levels at baseline, while all children with severe 25(OH)D deficiency belonged to the groups with no changes or worsening in ADOS scores. Therefore, 25(OH)D seems to be positively related to the response to probiotic treatment in improving ASD severity. Instead, having sufficient 25(OH)D levels does not affect the ADOS improvement in the placebo group, having all three groups have similar 25(OH)D mean levels, reinforcing the hypothesis of a synergistic effect between 25(OH)D and probiotics in subjects having adequate baseline 25(OH)D levels.

When the children were evaluated for GI symptoms, stratifying by treatment response (GI-Index worsened, GI-Index unchanged, and GI-Index improved), no significant differences in 25(OH)D values were observed among the three groups in our population. So, the negative correlation between 25(OH)D with the GI-Index could confirm that 25(OH)D deficiency or insufficiency could represent a pathological determinant for GI symptomatology, but not a crucial factor in determining the responsiveness to treatment with the probiotics.

All together, these data may suggest that the evaluation of 25(OH)D status before probiotic supplementation may be useful for predicting the response to treatment. In fact, in case of inadequate levels, a combined supplementation of 25(OH)D (targeting a blood concentration of at least 30 ng/mL) and probiotics could be considered to assist the probiotic response. Notably, all children with marked reduced 25(OH)D (<10 ng/mL) were in the group of worsened ADOS. Conversely, the percentage of children with 25(OH)D higher than 20 ng/mL resulted in 93% in the ADOS improvement group, and 56% in the ADOS unchanged/worsened group. Indeed, evidence of synergistic health effects of co-supplementation with 25(OH)D and probiotics is emerging in other clinical settings. In this framework, a recent study has suggested that the combined administration of *L. paracasei* DG with an oil-based cholecalciferol supplement could contribute to the maintenance of the adequate 25(OH)D serum levels in mice [48]. In addition to preclinical results, randomized controlled trials were recently conducted [49]. Abboud and coauthors in their systematic review of randomized controlled trials (six studies were double-blind, and once single-blind) supported the synergic effects of 25(OH)D and probiotics: conditions explored included schizophrenia, gestational diabetes, type 2 diabetes, coronary heart disease, polycystic ovarian syndrome, osteopenia, irritable bowel syndrome, and infantile colic. To the best of our knowledge, our study is the first exploring the relationship between 25(OH)D status on the effects of probiotic supplementation in ASD. At present, no studies have been carried out in subjects with ASD utilizing the combined administration of probiotic with 25(OH)D [50]. Nonetheless, Ghaderi and colleagues recently determined the effects of a novel combination of 25(OH)D and probiotic on metabolic and clinical symptoms in chronic schizophrenia, demonstrating beneficial effects not only on metabolic profiles, but also on the severity of psychiatric symptoms [51].

It has been shown that 25(OH)D is a factor that modifies the composition of the gut microbiota [45], demonstrating a potential reciprocal interaction between the gut microbiome and 25(OH)D. The synergic effect of probiotics with 25(OH)D could be due to the 25(OH)D effects at the gut level, involving immune cell differentiation, gut microbiota modulation, gene transcription, and gut barrier integrity [52,53]. Moreover, 25(OH)D and probiotic administration trigger a series of biochemical pathways that in turn reduce oxidative stress and inflammation and improve antioxidant defense implicated in brain function.

### 3.6. Strengths and Limitations

Although significant, R values of 0.25 or 0.34 are not high, thus, the confirmation of these associations is needed in future studies. Pharmacokinetic studies focused on the absorption and bioavailability of the supplements given to ASD children must be mandatory, rendering more precise (even personalized) the calculation of dosing regimens in future.

Moreover, although out of the focus of the present study but in view of paucity of data, it will be interesting to compare levels of 25(OH)D in ASD children with respect to typically developing (Italian) children of comparable ages and genders and/or siblings.

The strengths of the study include a relatively large sample size of patients, the two-arm design with a placebo, which allows for valid treatment group comparisons, the use of a battery of validated scores to assess ASD severity and GI symptoms, and the fact that patients act as their own controls, reducing the amount of error deriving from variance between individuals.

## 4. Materials and Methods

This study was carried out according to the standards for good ethical practice and with the guidelines of the Declaration of Helsinki. The study protocol was approved by the Pediatric Ethics Committee of the Tuscany Region (Approval Number: 126/2014) and substantial amendment (Approval Number 2–13/08/2019). Written informed consent from a parent/guardian of each participant was obtained.

### 4.1. Participants

Eighty-five ASD preschoolers were included in a double-blind, randomized controlled trial, funded by the Italian Ministry of Health and by Tuscany Region (grant GR-2011-02348280) on the efficacy of probiotic supplementation on GI, sensory, and core symptoms in ASD children [22]. Children were enrolled from November 2015 to February 2018 at the ASD Unit of the IRCCS Stella Maris Foundation (Pisa, Italy), a tertiary care university hospital. ASD diagnosis was performed by a senior child psychiatrist with specific expertise in clinical evaluation of ASD according to DSM-5 [1]. Exclusion criteria were brain anomalies; neurological syndromes/focal neurological signs; anamnesis of birth asphyxia, severe premature birth/perinatal injuries; epilepsy; significant sensory impairment; diagnosis of organic GI disorder or Coeliac Disease; and special diets. The probiotic supplement was De Simone Formulation, a patented mixture already approved for use in children (marketed as Vivomixx^®^ in EU, Visbiome^®^ in USA). The effects of probiotic supplementation vs placebo on GI, and ASD Core Symptoms have been previously published [47]. In 4 children, 25(OH)D blood levels were not assessed and were excluded by the analysis. Thus, baseline evaluation was conducted in 81 ASD children, and the response to a probiotic or placebo supplementation was studied in sixty-three children who completed the six months trial (placebo: n.32; probiotic: n.31), as measured by the change in the values of ADOS score for ASD severity and GI-index for GI symptoms [47].

### 4.2. ASD Severity

To assess ASD severity, we used the Total ADOS Calibrated Severity Score (ADOS-CSS) introduced in the Autism Diagnostic Observation Schedule-Second Edition (ADOS-2). The ADOS-2 [54] is a semi-structured assessment considered as the gold standard for the diagnosis of ASD with a demonstrated inter-rater reliability, test-retest reliability, and internal validity. The ADOS-CSS was created to standardize and compare ADOS-2 raw scores across different modules and ages. Calibrated scores are less influenced by the developmental functioning and demographics of the participant than raw totals and are therefore considered the best measure of core features of ASD in preschool children [55]. The ADOS-CSS is useful for comparing assessments across time and identifying trajectories of autism severity for clinical research [56]. ADOS-CSS can range on a scale-point from 1 to 10, while raw scores range from 0 to 28, with higher scores indicating greater severity.

### 4.3. GI Symptoms

The presence of GI symptoms was evaluated using a modified version of the GI Severity Index (GSI) [57] splitting the subjects into two groups (GI vs. No-GI). GSI is a 9 items-score to identify signs and symptoms of GI distress commonly reported by parents of children with ASD including nine variables. The first six variables (6GI-Index) evaluate specific GI symptoms (constipation, diarrhea, stool consistency, stool smell, flatulence, abdominal pain), and the additional three explore unexplained daytime irritability, night-time awakening, and abdominal tenderness (Total-GI-Index). A total score of 4 and above (with at least 3 score points from the first six items) are considered clinically significant for the classification of a subject within the GI group.

### 4.4. Blood Sample Collection and Analysis

A fasting blood sample (3 mL) was collected in ethylenediamine tetraacetic acid (EDTA) tube to perform the biomarkers quantitative analysis. Each tube was centrifuged for 10 min at 3500 rpm and all the plasma samples were stored at −80 °C until the bio-humoral analysis was performed. Cytokines were measured directly in the plasma through specific immunometric tests (MILLIPLEX MAP, human-magnetic bead panel, Millipore Corporation, Billerica, MA, USA) using an integrated multi-analyte detection platform (high-throughput technology Magpix system, Luminex xMAP technology, Luminex, Austin, TX, USA). This method allows to identify specific biomarkers (leptin, insulin, resistin, PAI-1, MCP-1, TNF-alfa, and IL-6) with some high level of automation and/or throughput. Magnetic Beads can make the process of automation and high throughput screening easier, receiving the advantage of ideal speed and sensitivity, allowing quantitative multiplex detection of analytes simultaneously. Each sample was analyzed in duplicate. In each experiment, a sample was analyzed as a quality control. Inter-assay variability was <10%.

Quantitative determination of 25(OH)D was performed by DiaSorin “LIAISON 25-OH Vitamin D TOTAL” CLIA, a direct competitive immunochemiluminescent assay, as previously described in detail [58]. In brief, the method does not require any pretreatment of samples (minimum sample requirement: 250 μL, measuring interval: 4–150 ng/mL, turn-around time: 40 min and assay throughput: 80 tests/h). During the first incubation phase, 25(OH)D is separated from its binding protein, and it interacts with binding sites on the solid phase. After the second incubation with the tracer, unbound material is washed off and a flash chemiluminescent signal generated by adding the starter reagents, then measured by a photomultiplier.

### 4.5. Statistical Analysis

The data are expressed as mean ± SD. Since some biomarkers (insulin, TNFalfa, IL-6, and resistin) were not normally distributed, we used log-transformation with parametric statistic tests. Data were back-transformed for result visualization. Statistical analysis included Student’s *t*-test (to determine the significance of the difference between the means of two data sets), χ2 tests to determine if there is a significant difference between predicted and observed frequencies in one or more categories of a contingency table, and linear regression. Moreover, unpaired analysis of variance (ANOVA) was used to evaluate whether an overall difference in the group data exists. In addition, a multivariate analysis was carried out to measure the relationships in which more than one independent variable (predictors) are related to the dependent variable.

Findings with *p* value < 0.05 were considered significant. StatView software (version 5.0.1; SAS Institute, Abacus Concept Inc., Berkeley, CA, USA) was used for data analyses.

## 5. Conclusions

ASD male children may be at a higher risk of 25(OH)D severe deficiency. The 25(OH)D status is inversely correlated with GI symptomatology. Moreover, the inverse correlation between 25(OH)D and leptin suggests that the maintenance of adequate levels of 25(OH)D may exert beneficial effects on the hormones, regulating the appetite and contributing to regular growth in ASD children.

The most important preliminary data that emerged from our study is that the beneficial response in ADOS Total Score to 6 months of probiotic administration is related to 25(OH)D status. Therefore, it may be of significance to evaluate through laboratory assessment 25(OH)D levels before starting a treatment with probiotics in ASD children, and to provide a vitamin D supplementation when needed, in order to reach a serum 25(OH)D target level of at least 30 ng/mL.

Thus, a co-administration of 25(OH)D and probiotics, in view of their possible synergistic effect could be considered as an effective supplementation in ASD children, and as such, merit further investigation in future studies.

Moreover, in addition to the administration together with probiotics, evaluation and supplementation with 25(OH)D could be considered in ASD from an early age, in view of its positive role on adverse GI symptoms and leptin levels.

## Figures and Tables

**Table 1:** Characteristics of the studied population.

Anthropometric Characteristics	
Number	81
Males	67 (83)
Females	14 (17)
Age (years)	4.1 ± 1.1
N-GI patients	54 (67)
GI patients	27 (33)
BMI (Kg/m^2^)	16.0 ± 1.7
**GI and ADOS values**	
Total GI severity score	3.6 ± 2.6
6GI-Severity score	2.0 ± 1.9
Social Affect (SA) ADOS CSS	6.5 ± 2.0
Restricted Repetitive Behaviours (RRB) ADOS_CSS	8.2 ± 1.4
Total ADOS_CSS	7.1 ± 1.8
**Blood parameters**	
25(OH)D (ng/mL)	24.8 ± 9.9
TNF-alfa (pg/mL)	6.1 ± 2.4
IL-6 (pg/mL)	6.2 ± 16.5
Leptin (ng/mL)	1.15 ± 0.9
Insulin (mU/L)	22.6 ± 10.5
Resistin (ng/mL)	23.4 ± 13.7
PAI-1 (ng/mL)	26.2 ± 19.2
MCP-1 (pg/mL)	127.8 ± 60.1

Values are n (%) or mean ± SD.

## Data Availability

The datasets generated and/or analyzed during the current study are not publicly available due the privacy policy (containing information that could compromise research participant privacy/consent) but are available from the corresponding author on reasonable request and with permission of parents of the involved children.
